# Hydrophobically Modified Chitosan in Biomedical Applications: Great Expectations vs. Translational Reality

**DOI:** 10.3390/polym18151849

**Published:** 2026-07-28

**Authors:** Agnieszka Piegat, Agata Goszczyńska, Agata Niemczyk

**Affiliations:** 1 Department of Polymer and Biomaterials Science, Faculty of Chemical Technology and Engineering, West Pomeranian University of Technology in Szczecin, 45 Piastow Ave, 70-311 Szczecin, Poland; agata.goszczynska@zut.edu.pl; 2 Department of Materials Technology, Faculty of Mechanical Engineering and Mechatronics, West Pomeranian University of Technology in Szczecin, 19 Piastow Ave, 70-310 Szczecin, Poland; agata.niemczyk@zut.edu.pl

**Keywords:** hydrophobically modified chitosan, biomaterial, chitosan modification

## Abstract

Hydrophobically modified chitosan has attracted considerable interest as an amphiphilic biomaterial for drug delivery, antimicrobial systems, and tissue engineering. Despite extensive research and promising biological performance, only a limited number of these materials have advanced toward clinically relevant technologies. This Perspective argues that the major barriers to translation are no longer related to biological efficacy but to insufficient reproducibility, inconsistent structural characterization, and limited attention to process engineering and scalable manufacturing. Rather than providing another comprehensive review, we discuss hydrophobically modified chitosan from a translational perspective, following its development from chemical modification and self-assembly to formulation engineering and representative biomedical applications. We highlight how molecular design, processing conditions, and material characterization collectively determine the reliability and practical applicability of these systems. Finally, we outline key priorities for future research, including standardized characterization protocols, harmonized experimental methodologies, and the implementation of engineering-oriented development strategies. We propose that future progress will depend less on introducing new chemical modifications and more on integrating polymer chemistry with process engineering to improve reproducibility and facilitate clinical translation.

## 1. Introduction

Chitosan is a partially deacetylated derivative of chitin whose combination of cationic character, biodegradability, and chemical versatility has made it one of the most extensively investigated polysaccharides for biomedical applications. Among its defining features are the presence of reactive amino and hydroxyl groups, which enable a wide range of chemical modifications, and a degree of deacetylation that strongly influences its physicochemical and biological behaviour. These intrinsic properties have established chitosan as a versatile platform for designing functional drug delivery systems and regenerative biomaterials [1].

Among the available modification strategies, grafting hydrophobic moieties onto the chitosan backbone has attracted particular attention because it fundamentally changes the behaviour of the polymer in aqueous media. Depending on the reactive functional groups present in the hydrophobic ligand and available on the chitosan backbone, conjugation may proceed through reductive amination, alkylation, or, in the case of fatty acids, carbodiimide-mediated *N*- or *O*-acylation, resulting in the formation of amide or ester bonds, respectively. The resulting amphiphilic derivatives combine the hydrophilic character of chitosan with hydrophobic domains capable of spontaneous self-assembly [2]. When using fatty acids, it is possible to perform *N*-acylation reactions involving amino groups (-NH_2_) or *O*-acylation reactions involving hydroxyl groups (-OH). Although *N*-acylation is generally favoured due to the higher reactivity of amino groups, preserving some cationic amino functionality remains important for maintaining the biological activity and mucoadhesive properties of chitosan. Selective *O*-acylation therefore represents an attractive alternative, although its practical implementation requires temporary protection of amino groups, increasing synthetic complexity and limiting translational applicability [3].

By grafting long-chain fatty acids onto the hydrophilic backbone of chitosan, usually through standard EDC/NHS coupling, one can create polymers that spontaneously form core–shell micelles when dissolved in water or water-rich environment [4,5]. The core of these micelles acts as a hydrophobic pocket that readily traps lipophilic drugs. At the same time, the outer chitosan shell keeps the whole system colloidally stable in solution. Since the shell retains its positive charge, it interacts strongly with mucosal linings and cellular membranes via mucoadhesion [6,7]. The self-assembly process usually occurs in an aqueous environment above the critical micelle concentration (CMC). This process, driven by the entropic gain resulting from minimizing contact between hydrophobic domains and water molecules, leads to the formation of unique supramolecular architectures, including spherical polymer micelles, nanocapsules, and core–shell aggregates. An additional advantage of this self-assembly process is that stable nanostructures can be generated without low-molecular-weight synthetic surfactants, many of which exhibit membrane-disrupting or cytotoxic effects [8,9]. Consequently, HMC provides a single polymer platform that simultaneously acts as the structural matrix, colloidal stabilizer, and bioactive interface. These materials have therefore been investigated not only as polymeric micelles but also as Pickering emulsion stabilizers [10,11], injectable hydrogels, antimicrobial coatings, and building blocks for more complex biomaterial architectures, including tissue-engineering scaffolds [12,13].

Importantly, self-assembly into micelles does not eliminate one of chitosan’s most valuable biological features—mucoadhesion. Because a substantial fraction of protonatable amino groups remains exposed within the hydrophilic shell, amphiphilic chitosan micelles preserve their ability to interact with mucosal surfaces and negatively charged cellular glycocalyx, extending residence time at the site of administration while enabling encapsulation of hydrophobic therapeutic agents.

**Figure 1:**
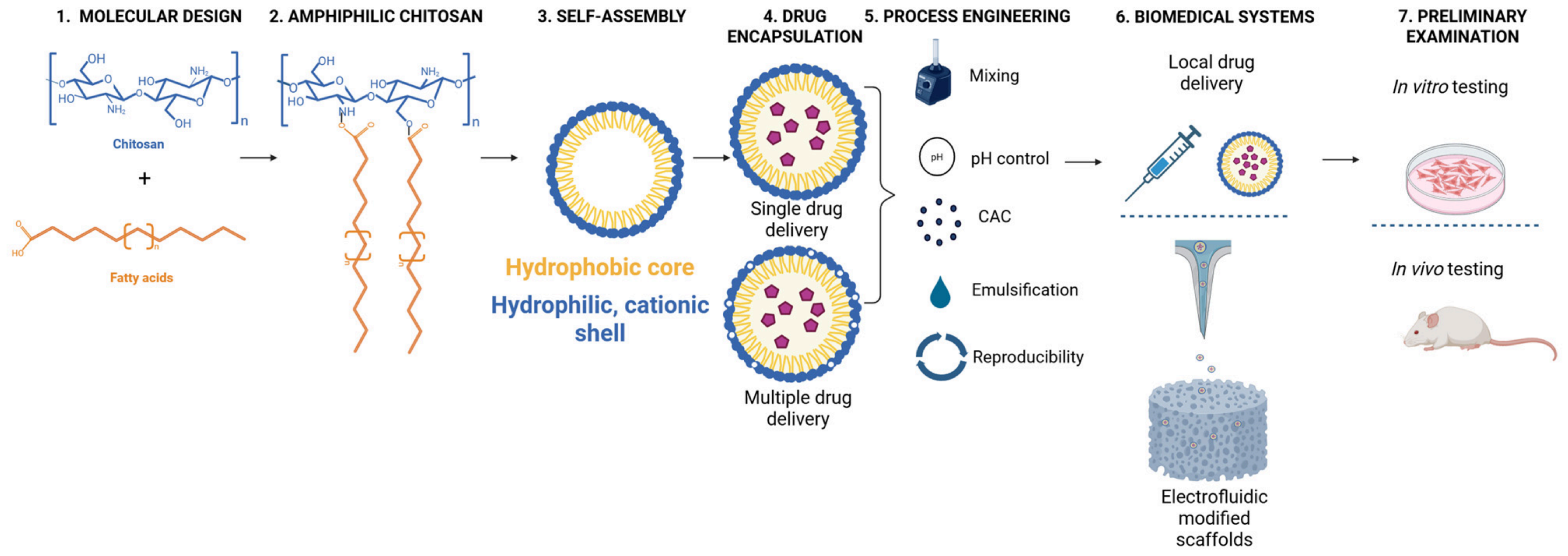
Translational pathway of fatty acid-modified chitosan from molecular design to biomedical applications. Created in BioRender. Piegat, A. https://BioRender.com/rrm682e, accessed on 29 June 2026. Schematic overview illustrating the evolution of hydrophobically modified chitosan from chemical modification and amphiphilic self-assembly into core–shell nanomicelles, through drug encapsulation and process engineering, toward biomedical applications.

Despite more than two decades of intensive research, only a small fraction of hydrophobically modified chitosan systems has progressed beyond laboratory-scale validation. This limited technology transfer is not primarily a consequence of insufficient biological performance but rather of inadequate control over material reproducibility, inconsistent characterization strategies, and the absence of scalable manufacturing protocols. The central argument of this Perspective is that successful translation depends on viewing hydrophobically modified chitosan not simply as a chemically modified polysaccharide, but as an engineering platform shaped by molecular design, process control, and manufacturing reproducibility. Accordingly, this discussion follows the complete development pathway: from reaction chemistry and structural verification, through self-assembled nanostructures and process optimization, to patented manufacturing solutions and three-dimensional biomaterial systems intended for regenerative medicine (Figure 1).

This Perspective is not intended to provide another comprehensive overview of hydrophobically modified chitosan or to summarize every reported biomedical application. Instead, it focuses on a question that has received comparatively little critical attention: why materials that consistently demonstrate promising biological performance so rarely progress toward reproducible technologies and clinically relevant products. By examining the field through the lens of translation rather than biological efficacy alone, the discussion aims to identify the engineering and methodological challenges that now represent the principal barriers to further development.

Rather than discussing these developments as isolated advances, this Perspective considers them as successive stages of a single translational pathway. Viewed together, these stages illustrate that successful translation depends on integrating synthetic chemistry, process engineering, structural characterization, and application-oriented material design rather than optimizing each element independently. The major challenge is therefore no longer demonstrating that hydrophobically modified chitosan works but understanding why promising laboratory systems still rarely evolve into reproducible technologies and clinically translatable biomaterials.

## 2. Recent Advances in Hydrophobically Modified Chitosan Nanostructures: From Material Discovery to Translational Design

Research on fatty acid-modified chitosan has evolved considerably over the past two decades. Early studies focused primarily on demonstrating the feasibility of amphiphilic modification and spontaneous self-assembly [14,15,16], whereas current work increasingly addresses how molecular architecture influences process reproducibility, colloidal stability, and ultimately translational potential [1,13,17]. This shift reflects a broader change in the field, from asking whether these materials can form nanostructures to understanding which structural and technological parameters determine their performance under pharmaceutically relevant conditions. Depending on their molecular design, amphiphilic chitosan derivatives spontaneously organize into polymeric micelles, nanoemulsions, or Pickering-type colloidal systems, each offering distinct opportunities and challenges for biomedical applications.

### 2.1. Polymeric Micelles: Synthesis, Critical Aggregation, and Lipophilic Payload Retention

The capacity of HMC to self-assemble into nanomicelles remains one of its most heavily exploited traits in target-specific drug delivery. Intramolecular and intermolecular hydrophobic interactions drive the segregation of the appended aliphatic fatty acid chains into an inner, dense lipophilic core, while the unreacted, hydrophilic glucosamine units extend outward to constitute a stabilizing cationic shell.

A review of recent literature highlights that the stability and thermodynamic viability of these systems are strictly bound to two variables: the length of the aliphatic fatty acid chain and the degree of substitution (DS) [18]. Saturated long-chain fatty acids (such as palmitic or stearic acid) significantly lower the critical micelle concentration (CMC) compared to their shorter counterparts. This drop in CMC is vital for intravenous translational applications; lower CMC values prevent premature micellar dissociation upon systemic dilution in blood plasma. Concurrently, introducing unsaturated acyl chains (e.g., oleic or linoleic acid) fundamentally alters core fluidity, which directly governs the encapsulation efficiency and release kinetics of hydrophobic molecules. From a translational perspective, these relationships are particularly important because they define formulation robustness rather than only drug-loading capacity. A micellar system with an exceptionally low CMC may exhibit excellent stability during systemic dilution, yet excessive hydrophobicity can simultaneously hinder drug release or compromise redispersion after storage. Consequently, optimizing fatty acid structure should be viewed as balancing manufacturing performance, storage stability, and therapeutic function rather than maximizing any single physicochemical parameter.

Taken together, these observations point to an important shift in the field. Early studies were largely concerned with demonstrating that HMC could self-assemble into stable nanostructures. Today, the more relevant question is not whether these systems can be formed, but whether they can be prepared reproducibly, maintain their properties during storage, and deliver consistent biological performance. In other words, the focus is gradually moving from material discovery to material reliability.

Recent studies illustrate a wide range of strategies for exploiting these amphiphilic domains. For instance, chemically modified chitosan bearing palmitoyl or oleoyl moieties have achieved outstanding success in packaging highly lipophilic, poorly water-soluble therapeutics. Saturated structures, such as *O*-palmitoyl chitosan, have been successfully used to enhance payload retention and permeability in lipid-based vesicle assemblies [19]. Similarly, low-molecular-weight chitosan modified with oleic acid exhibits an exceptionally low CMC (around 0.025 mg/mL), providing a hydrophobic reservoir capable of effectively encapsulating hydrophobic molecules like celecoxib or clarithromycin [4,6]. Despite this diversity of approaches, relatively few studies address how differences in synthesis, purification, or formulation influence the reproducibility of these systems, making direct comparison between reported results increasingly difficult.

However, a critical bottleneck in recent years is the precise control over payload retention versus trigger-responsive release. While a high DS ensures excellent encapsulation capacity for lipophilic models (such as α-tocopherol or ibuprofen), it often increases steric hindrance and rigidifies the core, slowing down drug diffusion. Molecular dynamics simulations and experimental benchmarks show that the flexible architecture of long-chain grafts facilitates a “sandwich-like” encapsulation mechanism, wrapping around the hydrophobic drug molecules to maximize entropic gains [20]. Despite these insights, engineering a micelle that exhibits zero-leakage during systemic circulation but prompt release at the target site remains a complex balancing act that is highly sensitive to the underlying chemical design.

Interestingly, a bibliometric overview of the last five years (2022–2026) reveals a noticeable plateau in the publication of straightforward studies dealing purely with lipophilic anticancer drug encapsulation inside binary fatty acid-chitosan micelles. Rather than reflecting a decline in interest, this trend suggests that the field has matured. Demonstrating simple drug loading is no longer the main objective. Instead, current efforts increasingly focus on achieving reproducible formulations that meet the practical requirements of pharmaceutical development. The contemporary bottleneck is no longer *if* these micelles can carry a therapeutic cargo, but *how* reproducible their structural and hydrodynamic properties are when subjected to strict pharmaceutical manufacturing parameters.

Bridging this translational gap requires greater attention to formulation engineering alongside polymer design. Similar challenges have been recognized across the broader field of polymeric nanomedicine, where reproducibility, process robustness, and scalable manufacturing are increasingly regarded as prerequisites for successful clinical translation rather than technical considerations. Recent reviews emphasize that formulation variables, including mixing conditions, solvent exchange, phase composition, and processing time, can substantially influence nanoparticle size, polydispersity, encapsulation efficiency, and colloidal stability, even when the polymer composition remains unchanged [21,22]. A similar conclusion has been reported for hydrophobically modified chitosan systems. Luna et al. demonstrated that systematic optimization of formulation variables, including solution pH and the crosslinker-to-polymer ratio during ionotropic gelation, significantly affected nanoparticle size, surface charge, stability, and encapsulation efficiency, highlighting that careful control of fabrication conditions is an essential component of HMC formulation [23]. Consequently, improving process control has become as important as developing new carrier chemistries, reflecting a broader shift from proof-of-concept formulations toward reproducible and manufacturable nanomedicines [24].

These processing parameters do considerably more than determine particle size. The hydrodynamic conditions established during emulsification influence how rapidly amphiphilic polymer chains reorganize at the oil–water interface, thereby controlling the extent to which hydrophobic fatty acid domains remain buried within the micellar core or become transiently exposed to the particle surface. This subtle structural rearrangement directly affects both colloidal stability and the affinity of the micelles for hydrophobic cargo. Insufficient mixing promotes heterogeneous core formation and premature drug leakage, whereas excessive shear may disturb the dynamic equilibrium of self-assembly. Optimizing mixing time, stirring speed, and phase ratio therefore represents an engineering strategy for controlling drug retention and release without introducing synthetic surfactants or altering the chemical composition of the polymer.

One example illustrating this engineering-oriented approach could also be our recently patented fabrication strategy (PL 248577 B1) [25]. Rather than relying on constant high-speed mixing, the protocol incorporates a controlled reduction in stirring rate (from 1000 to 750 rpm) during ionic gelation together with precisely regulated tripolyphosphate addition at pH 5.0. These seemingly modest process modifications substantially improved the reproducibility of nanoparticle formation and encapsulation efficiency while maintaining a stable particle size distribution across batches. Although this example represents a single formulation strategy, it illustrates a broader principle emerging across polymeric nanomedicine: overcoming translational barriers requires optimization of manufacturing parameters alongside molecular design, providing a practical bridge between laboratory formulation and scalable pharmaceutical production [21,24].

Taken together, these observations indicate that further advances in HMC are unlikely to arise from developing new amphiphilic derivatives alone. Instead, progress increasingly depends on integrating molecular design with formulation engineering, standardized characterization, and scalable manufacturing strategies.

### 2.2. Nanoemulsions and Multi-Phase Systems: Interfacial Dynamics and Morphology

Beyond single-component micellar aggregates, HMC has driven significant progress in multi-phase colloidal engineering. The amphiphilic nature of these macromolecules allows them to migrate toward oil–water interfaces, drastically lowering interfacial tension and acting as efficient macromolecular stabilizers in Pickering emulsions [10,11]. Unlike conventional low-molecular-weight surfactants, these polymers generate mechanically robust interfacial layers whose structure depends strongly on formulation conditions. Their significance extends beyond emulsion stabilization, as Pickering systems are increasingly recognized as surfactant-free platforms offering enhanced long-term stability, reduced toxicity, and improved control over interfacial phenomena.

Furthermore, the structural versatility of HMC has recently led to the discovery of unexpected morphological transitions that challenge traditional spherical assumptions. A notable breakthrough in multi-phase self-assembly involved the combination of HMC (modified with oleic, palmitic, or stearic acid) with α-cyclodextrin [26]. This connection induces the formation of non-spherical, hexagonally faceted platelets. The driving force behind this unique morphology is the host–guest inclusion complexation between the cyclodextrin cavities and the grafted alkyl chains of the chitosan backbone. This specific interaction forces the polymer into a crystalline arrangement that yields flat, sharp-edged micro- and nano-platelets, regardless of the precise length or unsaturation of the attached fatty acid [26]. Such structural control shows that HMC multi-phase systems can transition from fluid emulsion stabilizers to highly ordered, morphological templates for advanced biomaterial design. Importantly, these findings illustrate that controlling supramolecular organization extends beyond improving drug delivery. The same molecular interactions that govern micelle formation can also be exploited to generate ordered microarchitectures suitable for advanced biomaterials, providing a natural transition toward three-dimensional scaffold fabrication discussed in the following section. These examples also illustrate that the value of HMC extends beyond the development of new carrier systems.

Overall, the field appears to be entering a new stage of development. Although the design of new HMC derivatives continues to evolve, increasing attention is being directed toward producing materials with well-defined structures, reproducible formulation protocols, and predictable performance across laboratories and biomedical applications.

## 3. Translating Hydrophobically Modified Chitosan into Biomedical Systems: Lessons from Two Application Pathways

### 3.1. Hydrophobically Modified Chitosan Nanomicelles for Intraperitoneal Chemotherapy

Localized intraperitoneal chemotherapy represents one of the most demanding applications for advanced drug delivery systems because it requires prolonged residence within the peritoneal cavity while minimizing systemic exposure. Consequently, considerable research efforts have focused on developing polymeric nanoparticles, micelles, hydrogels, and other localized delivery platforms capable of improving intraperitoneal drug retention and therapeutic efficacy [27]. Recent systematic analyses indicate that these systems can enhance local drug accumulation while reducing systemic toxicity; however, further progress is still limited by challenges related to formulation reproducibility, manufacturing robustness, and clinical translation [27,28]. Among the various polymeric platforms under investigation, hydrophobically modified chitosan has attracted increasing attention because its amphiphilic architecture combines spontaneous self-assembly with mucoadhesion and the potential for prolonged local drug retention, making it particularly attractive for intraperitoneal treatment of advanced ovarian cancer.

When standard platinum drugs such as carboplatin or cisplatin are given intravenously, they rarely accumulate well in the peritoneum. Instead, patients end up facing severe systemic side effects, such as nephrotoxicity and profound bone marrow suppression, without getting sufficient amounts of the drug to the tumour site. Localized intraperitoneal (IP) chemotherapy is often more effective because it bathes the tumour metastases directly in high drug concentrations. The real issue, however, is that free small-molecule cytostatics do not stay in the cavity for long. Lymphatic drainage clears them out rapidly, which shortens the therapeutic window and forces clinicians to use repeated, highly stressful cycles of administration [29]. An improved understanding of the biology of metastatic cancer cell clusters within the peritoneal cavity has highlighted mucoadhesion as a promising mechanism for the selective binding of drug carriers to tumor cells. SK-OV-3 cells specifically secrete mucin type 1 (MUC1), a finding which has not been observed in endothelial cells of the peritoneal cavity and the external surface of internal organs [30]. These observations have stimulated the development of numerous mucoadhesive drug delivery systems designed to increase local residence time within the peritoneal cavity while improving selective interaction with tumour tissue. Accordingly, a variety of polymeric nanocarriers have been explored to enhance intraperitoneal drug retention and tumour targeting. For example, hyaluronic acid/poly(L-arginine) nanoparticles have been shown to prolong peritoneal residence and improve antitumour efficacy, illustrating that multiple polymer-based strategies are being developed to address the same translational challenge [31]. Taken together, these findings indicate that mucoadhesive carriers incorporating cytostatics represent a particularly promising strategy for improving localized intraperitoneal chemotherapy.

While biological targeting determines where the carrier should act, successful clinical translation also depends on the ability to manufacture formulations with consistent physicochemical properties and reproducible performance. Studies on HMC formulation have shown that optimizing polymer chemistry alone is insufficient to obtain reproducible drug carriers. Formulation parameters—including emulsification conditions and phase composition—can be equally important in determining micellar architecture, particle uniformity, and, ultimately, manufacturing reproducibility [32]. More broadly, these observations reflect a challenge that extends across polymeric nanomedicine, where robust formulation processes are increasingly recognized as being just as important as rational molecular design. Similar conclusions have been reported for amphiphilic polymeric nanocarriers based on block copolymers and lipid-based systems [33,34,35].

One example illustrating this engineering-oriented strategy is a recently developed carboplatin-loaded HMC micellar formulation designed to improve local intraperitoneal drug delivery. Preliminary observations suggest that careful control of formulation parameters enhances carrier stability and supports prolonged drug retention. Although these observations require further validation, they reinforce the broader concept that reproducible processing is as important as polymer chemistry for successful translation. Collectively, these observations illustrate that successful intraperitoneal drug delivery depends not only on selecting an appropriate carrier but also on integrating material design with robust formulation engineering and reproducible manufacturing strategies.

### 3.2. Polymeric Micelles for Combination Therapy Against Helicobacter pylori

The treatment of *Helicobacter pylori* infection has become increasingly challenging due to the limited stability of antibiotics in the acidic gastric environment, restricted penetration through the protective mucus layer, biofilm formation, and the global rise in antimicrobial resistance. Consequently, considerable research has focused on biomaterial-based drug delivery systems designed to improve local drug retention, protect therapeutics from degradation, provide controlled release, and enhance antibacterial efficacy [36,37]. Nanoparticles, microspheres, hydrogels, and mucoadhesive polymeric carriers are increasingly being investigated as promising platforms for overcoming these biological barriers and potentially improving eradication outcomes [36].

Among these biomaterial-based strategies, amphiphilic polymeric carriers have attracted increasing attention because they combine biocompatibility, mucoadhesion, controlled drug release, and the ability to self-assemble into nanostructured delivery systems [36,37]. The amphiphilic architecture of HMC drives their spontaneous self-assembly in aqueous or aqueous–alcoholic media. This core–shell organization may be particularly useful for designing combination therapies, as it can facilitate the simultaneous encapsulation of more than one therapeutic agent within a single vehicle. Depending on their physicochemical properties, hydrophobic substances can be solubilized inside the lipophilic fatty acid core, while hydrophilic or charged molecules can associate with the outer cationic chitosan shell.

Oral eradication of *Helicobacter pylori* presents a different translational challenge. Unlike intraperitoneal chemotherapy, the formulation must remain stable within the dynamic gastric environment while simultaneously delivering chemically distinct therapeutic agents. The amphiphilic architecture of hydrophobically modified chitosan enables this dual functionality by accommodating hydrophobic compounds within the micellar core and hydrophilic drugs within the surrounding polymer shell. Standard treatment requires a multi-drug regimen combining direct-acting antibiotics, such as amoxicillin (AMX), with proton pump inhibitors like lansoprazole (LANSO) to control local pH and preserve antibiotic stability. The structural stability of HMC micelles may provide a suitable platform for co-encapsulating both drugs within a single delivery system. Within this broader group of biomaterial-based carriers, hydrophobically modified chitosan offers an attractive platform for combination therapy because it enables the simultaneous incorporation of compounds with markedly different physicochemical properties [36]. Given their distinct chemical properties, we hypothesized that the water-soluble AMX resides primarily within the outer chitosan shell, whereas the lipophilic LANSO remains molecularly shielded inside the hydrophobic core.

Preliminary biological evaluation suggests that amphiphilic HMC systems may facilitate combination therapy while partially mitigating local irritation associated with free-drug administration. These findings remain exploratory but illustrate how carrier architecture may simultaneously influence drug delivery and host response.

Importantly, when tested against a reference strain and four clinical isolates of *H. pylori*, the dual-loaded nanomicelles showed enhanced eradication efficiency compared to single-drug systems. This enhanced efficacy is unlikely to result solely from the combined release of AMX and LANSO and may also reflect the intrinsic antimicrobial properties of the chitosan-based carrier. Previous studies demonstrated that *N*,*O*-acylated chitosan derivatives, particularly those modified with linoleic acid, possess notable antibacterial activity that is highly dependent on environmental pH [38]. Within the pH range of 5.0 to 6.0—which aligns with the microenvironment established during *H. pylori* therapy—the HMC carrier is expected to retain antibacterial activity against bacterial membranes. By combining the inherent pathogen-disrupting capability of the fatty acid-modified chitosan backbone with the localized, shielded delivery of two complementary drugs, these systems may provide complementary therapeutic benefits at the mucosal barrier. At the same time, the complex immunological response observed in vivo highlights the need for further structural optimization before clinical translation can be realistically considered. These findings are consistent with the broader trend toward application-specific design of amphiphilic polysaccharide carriers rather than universal drug delivery platforms [39,40]. More broadly, these observations are consistent with current trends in nanomedicine, where multifunctional carriers are increasingly designed to combine prolonged gastric residence, controlled drug release, and improved interaction with the mucus layer to enhance local therapeutic efficacy [37,41].

Thus, hydrophobically modified chitosan may therefore be viewed not as an isolated material platform but as part of the broader evolution of multifunctional mucoadhesive nanocarriers designed to improve localized therapy of *H. pylori* infection.

### 3.3. Electrofluidic Processing: Integrating Hydrophobically Modified Chitosan into Hierarchical Scaffolds

Recent advances in electrohydrodynamic processing have increasingly combined electrospinning and electrospraying to fabricate hierarchical multifunctional biomaterials. While electrospinning provides a fibrous structural framework that mimics the extracellular matrix, electrospraying enables the controlled deposition of diverse functional components onto pre-formed scaffolds, thereby introducing localized biological functionality without compromising the structural integrity of the construct [42,43].

A wide range of materials has been incorporated into electrospun scaffolds using electrospraying, including polymeric nanoparticles, hydroxyapatite particles, growth factors, genes, enzymes, and cartilage-specific biomolecules, highlighting the versatility of this surface-functionalization strategy [43]. Within this broader context, hydrophobically modified chitosan may also be adapted for electrospray-based surface functionalization of electrospun scaffolds. Accordingly, HMC may find applications beyond colloidal drug delivery by integrating self-assembled nanoparticles with electrofluidically fabricated scaffolds. Rather than acting as the structural component of a scaffold, HMC can be electrosprayed as self-assembled nanoparticles onto electrospun fibres and three-dimensional printed architectures, introducing localized drug-delivery functionality while preserving the mechanical integrity of the construct.

In our work, hierarchical scaffolds were fabricated by combining melt-extrusion 3D printing, electrospinning of poly(ε-caprolactone) fibres, and electrospraying of linoleic acid-grafted chitosan. The principal challenge was not the fabrication of the hierarchical scaffold but the optimization of HMC electrospraying, since this amphiphilic derivative had not previously been processed using this technique. Owing to its self-assembling nature, it required careful adjustment of the electrospraying process.

The optimized process yielded homogeneous HMC nanoparticles uniformly deposited on both the electrospun fibre network and the 3D-printed struts, producing a hierarchical scaffold with distinct structural and functional compartments. While the electrospun fibres improved cell seeding by increasing the available adhesion area, the HMC nanoparticles maintained favourable cell adhesion while providing a versatile platform for the future incorporation of therapeutic molecules. These findings illustrate how the self-assembly behaviour of hydrophobically modified chitosan can be translated from nanocarrier design to the surface functionalization of tissue-engineering scaffolds. More broadly, this example illustrates how electrofluidic processing may broaden the application scope of HMC beyond drug delivery into multifunctional biomaterial platforms [44].

## 4. Toward Reproducible and Translatable Hydrophobically Modified Chitosans

**Figure 2:**
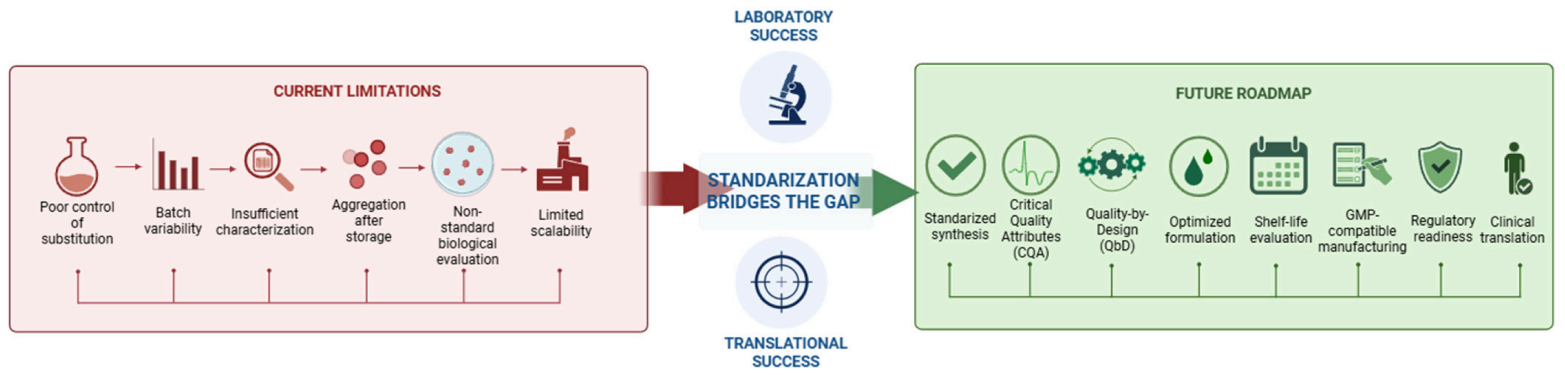
From laboratory success to translational reality: bottlenecks and future roadmap for hydrophobically modified chitosan. Created in BioRender. Piegat, A. https://BioRender.com/dgluze6, accessed on 26 June 2026. Conceptual summary of the major barriers currently limiting the clinical translation of hydrophobically modified chitosans together with the proposed engineering-oriented roadmap required to achieve reproducible, scalable, and clinically relevant biomaterial systems.

Current evidence suggests that the major challenges facing hydrophobically modified chitosan are shifting from demonstrating biological activity toward improving reproducibility, standardization, and manufacturing robustness. Although many studies have confirmed the therapeutic potential of these materials, comparatively less attention has been paid to methodological issues that determine whether a promising laboratory formulation can be reproduced, scaled, and ultimately translated into clinical use. Against this background, several recurring bottlenecks can be identified, providing a framework for discussing the key barriers to reproducible and clinically translatable HMC systems (Figure 2).

### 4.1. Chemical and Structural Bottlenecks: Misinterpreted Modifications

One of the major barriers to the translation of HMC is its limited structural reproducibility, particularly regarding the degree of substitution of grafted fatty acid chains. Minor fluctuations in DS can substantially influence the CMC, hydrophobic domain exposure, and subsequent in vivo thermodynamic stability. However, achieving a predictable DS on a larger scale is consistently thwarted by a critical, yet frequently overlooked synthetic issue: the actual chemical path of the acylation reaction.

When utilizing standard carbodiimide coupling systems, such as EDC/NHS, modification is frequently interpreted primarily in terms of amide bond formation at the primary amino groups of D-glucosamine units, and the resulting products are commonly described as *N*-acylated chitosan derivatives in the literature [45,46]. Experimental studies indicate that simultaneous *N*- and *O*-acylation may occur under typical reaction conditions, contributing to structural heterogeneity and potentially affecting surface charge, self-assembly behaviour, and biological performance. Moreover, mechanistic studies of carbodiimide-mediated coupling reactions have demonstrated that activated carboxyl groups may also react with hydroxyl functionalities to form ester linkages, indicating that exclusive amide formation should not be assumed a priori [47]. This secondary *O*-acylation primarily affects the C-3 and C-6 hydroxyl groups of the chitosan backbone, further increasing structural heterogeneity. The relative contribution of *N*- and *O*-acylation is influenced by the coupling system, pH, acid identity, and reaction medium. In the linoleic acid–chitosan system, reactions conducted in 1 wt% acetic acid at pH 4.7, acidified acetic acid at pH 4.0, and aqueous HCl at pH 4.0 were compared using either EDC or EDC/NHS [48,49]. The EDC/NHS systems increased the relative contribution of *N*-acylation compared with EDC alone, although none of the investigated conditions provided complete chemoselectivity. Consequently, direct coupling with unprotected chitosan cannot be assumed to yield exclusively *N*-acylated derivatives. Selective *O*-acylation generally requires minimizing competing *N*-acylation by reducing the nucleophilicity of amino groups, either through temporary *N*-protection, such as *N*-phthaloylation, or through strong protonation in an acidic reaction medium [50]. In the protection-based approach, acylation of the available hydroxyl group is followed by removal of the protecting group [51].

Overlooking this dual reactivity may lead to an incomplete interpretation of the polymer’s spatial conformation, surface charge density, and chemical architecture, thereby reducing the predictability of its biological performance. Consequently, reporting only the nominal fatty acid content or the overall degree of substitution is insufficient for meaningful comparison between studies. Without identifying how substituents are distributed between amino and hydroxyl groups, materials with apparently similar degrees of substitution may exhibit markedly different colloidal behaviour, surface charge, drug-loading capacity, and biological performance. Structural reporting should therefore extend beyond quantitative substitution and include the nature of the substitution pattern itself. Where possible, complementary analytical techniques capable of distinguishing substitution at amino and hydroxyl groups should therefore be incorporated into structural characterization protocols.

Many of these limitations are not unique to HMC but reflect broader challenges associated with chitosan as a raw material. As highlighted by Bellich et al. in their critical analysis “The Good, the Bad and the Ugly of Chitosans”, inconsistencies in reporting the degree of acetylation, molecular weight distribution, raw material origin, and impurity profile continue to compromise the reproducibility of chitosan-based biomaterials [52]. Hydrophobic modification introduces an additional level of structural complexity, making rigorous characterization even more essential. The possibility of multiple substitution pathways further increases this complexity, emphasizing the need for standardized structural characterization rather than reliance on nominal substitution values alone. Comparable challenges have been recognized for other chemically modified polysaccharides, where small differences in substitution patterns may translate into substantial changes in physicochemical behaviour and biological performance [53,54,55]. Consequently, improving structural reproducibility should be viewed as a broader challenge in polysaccharide engineering rather than a limitation unique to HMC.

### 4.2. Formulation Engineering: The Overlooked Physical Gaps

Even well-designed HMCs can lose their original physicochemical properties during purification, freeze-drying, storage, or reconstitution if these steps are not carefully optimized. Although these processes are often treated as secondary to polymer synthesis, they can have a profound influence on colloidal stability and, consequently, biological performance. Among the post-processing variables, buffer composition provides a representative example of how seemingly minor formulation choices can markedly influence the final physicochemical properties of HMC systems. Although this observation should be validated across a broader range of hydrophobically modified chitosan formulations, it illustrates a broader principle: post-processing conditions deserve the same level of attention as polymer synthesis when robust and reproducible HMC-based drug delivery systems are being designed. This challenge is not unique to HMC systems. For example, the clinically advanced core-crosslinked polymeric micelle formulation CPC634 required dedicated optimization of freeze-drying, cryoprotection, residual moisture content, and reconstitution to eliminate dependence on an ultra-low-temperature cold chain while preserving particle size, morphology, and drug-release characteristics [56]. Such studies illustrate that formulation engineering extends well beyond nanoparticle synthesis and becomes an integral component of successful translation.

Based on these observations, several practical aspects deserve routine consideration during formulation development:

Control of Physicochemical Environment: The self-assembly and charge-mediated stabilization of HMC micelles are highly sensitive to pH fluctuations. Hence, synthesis and micellar preparation must be maintained within a strict, narrow pH window (typically around pH 4.5–5.0).

Temperature and Cooling Control: Micelle formation is a thermodynamically driven process dependent on chain mobility. Consequently, temperature-controlled centrifugation (e.g., at approximately 4 °C) may be beneficial for preserving the structural integrity of hydrophobic domains and minimizing aggregation during isolation.

Immediate Post-Synthetic Lyophilization: Immediate freeze-drying after dialysis and purification appears to preserve the porous structural organization of HMC, facilitating subsequent redispersion and improving batch-to-batch reproducibility.

Collectively, these observations suggest that formulation engineering appears to be an integral part of HMC design rather than a downstream technical step. Standardizing post-synthetic processing may be as important as controlling polymer chemistry for achieving reproducible physicochemical properties and facilitating future translation.

### 4.3. Biological Characterization: Toward More Predictive Preclinical Evaluation

Preclinical evaluation of HMC is also influenced by methodological inconsistencies, particularly in immunological and antimicrobial screening, which may complicate comparisons between studies and hinder translational assessment. In terms of biocompatibility, many studies rely on short-term in vitro cell viability assays that fail to capture complex in vivo tissue responses. Although such assays provide valuable initial information, they offer only a limited prediction of in vivo biological performance, where inflammatory responses, immune modulation, and tissue-specific interactions become equally important determinants of translational success [57,58]. Current evidence suggests that cytocompatibility should be evaluated as a concentration-dependent phenomenon while also considering interactions between acylated HMCs and the host immune system. Experimental studies in murine models suggest that although HMCs generally exhibit acceptable biocompatibility, certain fatty acid derivatives may induce localized mucosal or cellular irritation at higher concentrations. These observations emphasize that demonstrating low in vitro cytotoxicity alone should not be regarded as sufficient evidence of biological safety for future clinical applications [59].

Another challenge that arises when developing HMC as an antibacterial material against pathogens such as *Helicobacter pylori* is selecting appropriate microbiological assays [60]. A vast number of papers continue to report on the antimicrobial potential of HMC using classic agar-diffusion methods, such as disc diffusion or agar-well assays. Studies show that diffusion-based assays provide limited information for evaluating amphiphilic polymeric systems because restricted diffusion through agar can mask genuine antimicrobial activity [61]. This limitation is not unique to HMC but reflects a broader methodological challenge encountered during the evaluation of polymeric and nanoparticle-based antimicrobial materials. Selecting microbiological assays that are compatible with the physicochemical properties of the investigated system is therefore essential for obtaining meaningful and comparable biological data. Standardization of testing protocols would also facilitate comparison between studies and improve the translational assessment of newly developed polymeric antimicrobial systems [60,62]. For this reason, liquid-phase assays generally provide a more reliable assessment of the biological performance of hydrophobically modified chitosan [38,60].

### 4.4. A Roadmap to the Clinic: Standardizing the Future of HMC

Rather than being limited by the availability of new chemical modifications, further progress is increasingly constrained by issues of reproducibility, characterization, and manufacturability that are widely recognized across polymeric nanomedicine. Drawing on broader experience from translational biomaterials research, several methodological priorities emerge that are also directly applicable to hydrophobically modified chitosan.

- **Correlative Synthesis and Structural Verification Standards**: Reliable comparison between HMC systems requires standardized reporting of synthesis conditions together with rigorous structural characterization [63]. Synthesis parameters, including reaction chemistry and processing conditions, should be reported together with relevant material attributes, particularly DS and the *N*/*O* substitution pattern, because these parameters directly influence self-assembly, drug loading, surface charge, and biological performance [64]. Quantitative ^1^H NMR can be used for reliable overall DS determination because, under validated quantitative acquisition conditions, signal integrals are directly related to the numbers of contributing nuclei. For amphiphilic HMC derivatives, aggregation-related shortening of transverse relaxation times may cause severe signal broadening and incomplete detection of the polymer, thereby biasing the calculated DS. Sample preparation and acquisition conditions should therefore allow quantitative observations of both the polymer backbone and substituent signals. Complementary 2D NMR methods, particularly ^1^H-^13^C HMQC, can help identify substitution in amino and hydroxyl groups [49,65].
- **Liquid-Phase Antimicrobial Standard:** Antimicrobial evaluation would benefit from greater methodological consistency, with quantitative susceptibility assays, such as broth microdilution performed according to CLSI or EUCAST recommendations, serving as the primary basis for comparison whenever applicable [60]. While agar diffusion assays remain useful for preliminary screening, their interpretation may be less reliable for nanoparticle-based systems because limited material diffusion can underestimate antimicrobial activity [61]. Complementing susceptibility testing with assays that probe membrane integrity, metabolic activity, or bacterial viability could provide a more comprehensive understanding of the mechanisms underlying HMC antimicrobial performance.
- **Long-Term Stability and Shelf-Life Testing:** Long-term evaluation of the physical and chemical stability of HMC formulations should become a routine component of preclinical characterization. Monitoring parameters such as hydrodynamic diameter, colloidal integrity, and cargo retention during storage would improve confidence in robust formulation and facilitate comparisons between independently developed systems [63,65]. Despite their importance for pharmaceutical development and regulatory translation, systematic shelf-life studies remain relatively uncommon in HMC literature.
- **Targeted Formulation Buffering:** The physicochemical environment in which HMC formulations are prepared and stored, including buffer composition, pH, and ionic strength, should be considered an integral part of formulation design rather than a secondary optimization step. Careful control of these parameters can improve colloidal stability, minimize aggregation during storage, and support consistent redispersion and biological performance [63,66]. Incorporating formulation conditions into early-stage development would also facilitate reproducibility and more meaningful comparisons across independently developed HMC systems.

Looking ahead, successful translation of HMC will depend not only on advances in material design but also on how these materials are developed and manufactured. In recent years, pharmaceutical development has increasingly adopted the Quality-by-Design (QbD) concept, which emphasizes understanding how material attributes and processing parameters influence the quality and reproducibility of the final product [67,68,69]. Applying QbD principles to HMC manufacturing requires defining a quality target product profile (QTPP) based on the intended application and route of administration and identifying the associated critical quality attributes (CQAs). Depending on the intended product, relevant CQAs may include DS and the *N*/*O* substitution pattern, molecular weight distribution, residual reagents and solvents, microbiological quality, and storage stability. For drug-delivery formulations, particle size, polydispersity, surface charge, drug loading, and the drug release profile may also represent relevant CQAs. Potential critical material attributes of the starting chitosan, particularly its molecular weight distribution and degree of deacetylation, and critical process parameters, such as pH, reagent stoichiometry, reaction medium, temperature, reaction time, purification, drying, and, where applicable, sterilization, should be identified and prioritized through risk assessment. Their individual and combined effects on the CQAs can subsequently be investigated using design of experiments.

**Figure 3:**
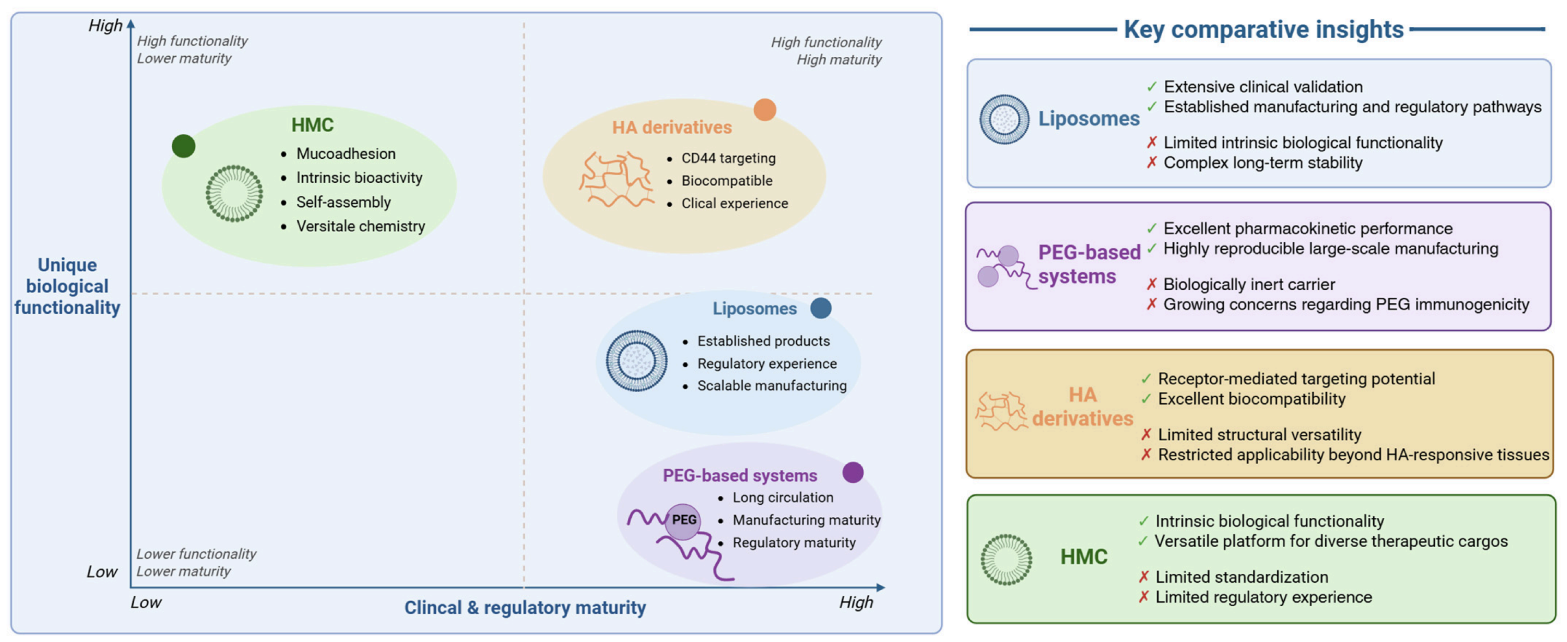
Positioning hydrophobically modified chitosan in the nanocarrier landscape. Created in BioRender. Piegat, A. https://BioRender.com/2kvrutb, accessed on 26 June 2026. A comperative view of clinical maturity and functional potential of selected nanocarrier platforms.

Hydrophobically modified chitosan can be viewed within the broader landscape of amphiphilic polymer platforms currently under development for biomedical applications [35]. Among natural polymers, amphiphilic hyaluronic acid derivatives provide perhaps the closest comparison, combining self-assembly with receptor-mediated targeting and excellent biocompatibility. However, unlike chitosan, they do not inherently offer mucoadhesive or antimicrobial properties. At the other end of the spectrum, mPEG-based and liposome nanocarriers remain the most clinically advanced platform, benefiting from well-established manufacturing strategies and extensive regulatory experience [70]. In this context, HMC may be viewed not as a replacement for existing systems but as a complementary platform with advantages in applications where mucoadhesion, intrinsic bioactivity, and structural versatility are all desirable (Figure 3, Created in BioRender. Piegat, A. https://BioRender.com/2kvrutb, accessed on 22 July 2026).

Taken together, these recommendations do not represent a new synthetic strategy but rather a framework for improving the quality and reproducibility of future research. Progress toward clinical translation will depend on integrating standardized material characterization, rational process engineering, pharmaceutical development principles, and application-oriented validation.

## 5. Conclusions

Hydrophobically modified chitosan has progressed far beyond the stage of demonstrating proof-of-concept biological activity. Over the past two decades, advances in synthetic chemistry, self-assembled nanostructures, formulation engineering, and electrofluidic processing have established these materials as versatile platforms for drug delivery and regenerative medicine. The challenge facing the field is therefore shifting from demonstrating biological functionality toward achieving reproducible manufacturing and clinically relevant translation.

The evidence discussed throughout this Perspective suggests that successful translation depends on integrating four complementary levels of development: rational molecular design, rigorous structural characterization, controlled formulation engineering, and scalable manufacturing. Improvements at only one of these levels are unlikely to overcome the persistent gap between promising laboratory results and practical biomedical applications. Instead, reproducibility must become a primary design criterion from the earliest stages of material development.

HMC also exemplifies a broader challenge shared by many natural polymer-based biomaterials. As previously highlighted for chitosan itself, variability in raw materials, incomplete physicochemical characterization, and inconsistent experimental methodologies continue to limit meaningful comparison between studies. Hydrophobic modification further increases this complexity and reinforces the need for standardized analytical protocols, transparent reporting of structural parameters, and harmonized experimental practices.

The next stage of development is therefore unlikely to be driven by another generation of chemical modifications, but rather by harmonizing experimental methodologies across laboratories and strengthening the connection between chemistry, process engineering, and application-driven material design. Together, these efforts provide a realistic pathway for transforming hydrophobically modified chitosan from academically successful materials into reproducible biomedical technologies with greater potential for pharmaceutical and regenerative medicine applications.

## Data Availability

No new data were created or analyzed in this study. Data sharing is not applicable to this article.
